# P-561. Long-Acting Cabotegravir and Rilpivirine in HIV Individuals Over 60 years: a Real-World Study (RELATIVITY Cohort)

**DOI:** 10.1093/ofid/ofae631.760

**Published:** 2025-01-29

**Authors:** Jesús Troya, Enrique Bernal, Luis Morano, Marisa Montes, María José Galindo, María José Crusells, Miguel Torralba, Mireia Santacreu, Juan Emilio Losa, Miguel de Zárraga, María Aguilera, Noemí Cabello, Patricia Martín, Mar Masiá, Alfonso Cabello, María Antonia Sepúlveda, Maria del Carmen Fariñas, Alberto Diaz-De Santiago, María Jesús Vivancos, Jara Llenas, María del Carmen Montero, Beatriz de la Calle, Ruth Calderón, María del Mar García, Álvaro Cecilio, Roberto Pedrero Tomé, Luis Buzón-Martín

**Affiliations:** Hospital Universitario Infanta Leonor, Madrid, Madrid, Spain; Reina Sofía General University Hospital, Murcia, Murcia, Spain; Hospital Universitario Álvaro Cunqueiro, Vigo, Galicia, Spain; Hospital Universitario La Paz, Madrid, Madrid, Spain; Hospital Clínico de Valencia, Valencia, Comunidad Valenciana, Spain; Hospital Clínico Universitario Lozano Bless, Zaragoza, Aragon, Spain; Hospital Universitario de Guadalajara, Guadalajara, Madrid, Spain; Hospital Universitario 12 de Octubre, Madrid, Madrid, Spain; Hospital Universitario de Alcorcón, Alcorcón, Madrid, Spain; Hospital Sán Agustín, Avilés, Asturias, Spain; Hospital Universitario de la Princesa, Madrid, Madrid, Spain; Hospital Clínico, madrid, Madrid, Spain; Hospital de Denia Marina Salud, Alicante, Comunidad Valenciana, Spain; Hospital General Universitario de Elche, Elche, Comunidad Valenciana, Spain; Hospital Fundación Jiménez Díaz, Madrid, Madrid, Spain; Hospital Virgen de la Salud, Toledo, Castilla-La Mancha, Spain; Hospital Universitario Marqués de Valdecilla, Santander, Cantabria, Spain; Puerta de Hierro University Hospital, Madrid, Madrid, Spain; Hospital Universitario Ramón y Cajal, Madrid, Madrid, Spain; Hospital de la Vega Baja, Alicante, Comunidad Valenciana, Spain; Hospital de Torrejón, Torrejón, Madrid, Spain; Hospital Nuestra Señora del Prado, Talavera, Castilla-La Mancha, Spain; Hospital Universitario de Fuenlabrada, Fuenlabrada, Madrid, Spain; Hospital Universitario de Vinalopo, Alicante, Comunidad Valenciana, Spain; Hospital Universitario Miguel Servet, Zaragoza, Aragon, Spain; Hospital Universitario Infanta Leonor, Madrid, Madrid, Spain; Hospital Universitario de Burgos, burgos, Castilla y Leon, Spain

## Abstract

**Background:**

The switching strategy to long-acting cabotegravir and rilpivirine (CAB+RPV) has emerged as a standard approach for people living with HIV (PLWH), offering high efficacy, safety, and convenience rates. Nevertheless, there is a scarcity of data regarding older PLWH, an important and growing population with physiological differences and arising comorbidities.

**Methods:**

We conducted a multicenter, non-controlled, retrospective study on HIV virologically suppressed individuals who switched to long-acting CAB+RPV. We evaluated demographic and clinical factors associated with this switch in individuals over 60 years of age.

**Results:**

The study included 154 individuals from 27 hospitals in Spain, representing 12.8% of the Relativity cohort, which comprised 1204 individuals. The median age was 63 years (range: 61 to 68), with 77.9% being men. 89.5% were Spaniards, followed by South Americans (6.5%). HIV transmission ocurred via HSH in 42.6% of cases and heterosexual in 27.7%. Comorbidities were present in 70.1% of individuals, with 17.5% having three and 3.9% having four. The most prevalent comorbidities were dyslipidemia (45.5%), high blood pressure (32.5%), and osteoporosis (19.5%).

The HIV infection median time was 22 years (range: 13 to 31), the antiretroviral therapy median time was 18 years (range: 11 to 24), and the median time of viral suppression was 12 years (range: 8 to 17). 24.5% were in CDC stage C3. The nadir CD4+ cell count was 240 (range: 111 to 374) cells/mm³, and 5.8% had previous virological failure with resistance mutations in 100% (NRTI, PIs or both). ART before switching included DTG/3TC (31.8%), DTG/RPV (28.6%), BIC/FTC/TAF (16.2%), DRV/c/FTC/TAF (5.2%), and EFV/FTC/TDF (1.9%). The main reasons for switching were patient request (43.5%), improvement in quality of life (35.7%), and simplification (23.4%). The efficacy rate at week 28 was 100%, with no discontinuations.

**Conclusion:**

In a real-life setting, switching to long-acting CAB+RPV proves to be a viable option for individuals over 60 years old, with long-standing HIV infection and a high burden of baseline comorbidities, demonstrating sustained virological control over the initial 28 weeks of this treatment. Therefore, it should also be offered to older patients, even if they have multiple treated comorbidities.

**Disclosures:**

**All Authors**: No reported disclosures

